# Assessment of knowledge on emergency contraceptives and factors associated with utilization among female students in Bonga College of Education, Southwest Region, Ethiopia: cross-sectional study

**DOI:** 10.1186/s12909-024-05535-7

**Published:** 2024-05-15

**Authors:** Ketema Deribew

**Affiliations:** Department of Biology, Bonga College of Education, Bonga, Ethiopia

**Keywords:** Emergency contraception, Bonga, Ethiopia

## Abstract

**Background:**

Unintended pregnancy is a major public health problem in sexually active female students in Ethiopia. In higher education, female students are exposed to unprotected sex and are at risk of pregnancy, abortion, and its associated problems.

**Objective:**

The objective of this study was to assess knowledge of female students about emergency contraceptives and determine factors associated with utilization among college female students at Bonga College of Education, Southwest Ethiopia.

**Methods:**

The study was conducted from November 10, 2022 to May 30, 2023. All female students of Bonga College of education in all departments were included in this study purposively. Data were collected using Amharic version pretested questionnaire. Data obtained from the survey was entered into Microsoft Excel 2010 and analysed with SPSS version 20.0. Data summary was done with descriptive statistics. Logistic regression was used to measure associations between dependent and independent variables. Odds ratio was used to measure strengths of association between variables. Statistical significance was considered at 95% confidence level (CL). *P*-value less than 0.05 was considered significant during the analysis.

**Results:**

In this study a total of 103 College female students were involved. The mean age of the respondents was 20.6 (SD ± 2.06) years. The finding showed that 31 (31.1%) female students had started sexual intercourse and among them 58.1% faced pregnancy. Among the total sexually experienced respondents, 93.5% use contraceptive methods while others 6.5% do not use. Among the total 31 study participants, 27(87.1%) started using EC. The majority of pregnancy (83.3%) was intended type whereas 16.7% was unwanted pregnancy. Regarding the general knowledge about contraceptive methods, 19(18.4%) had poor knowledge. Among the total 103 female college students, 66(64.1%) heard about emergency contraceptives. Forced sex and unprotected free sex are predicting factors that induces female students to use emergency contraceptives. Fear of discontinuing school was the main inducing factor to commit abortion. Logistic regression analysis showed that college female students whose age category above 25 years were more likely to use emergency contraceptives. Students who came from urban area are more likely to use EC than rural areas. Married female students (AOR = 2.5, 95% CI: 0.76, 8.7) were two times likely to use EC as contraceptive method.

**Conclusions:**

Female students who came from urban area use EC better than who came from rural areas. Majority of sexually active female students had good practice and knowledge of using EC but some had poor knowledge. Forced sex and free sexual practice are key determinant factors that induces to use EC. Abortion was mainly done in private clinic. Fear of discontinuing school was determinant factors identified to commit abortion. Therefore, responsible bodies should develop strategies to improve female students’ reproductive health related to emergency contraceptives.

## Introduction

In the past two decades, there has been a significant growth in the number of women who want to utilize family planning, from 900 million in 2000 to approximately 1.1 billion in 2020. Consequently, women who are using contraceptive method increased from 663 to 851 million and the contraceptive prevalence rate increased from 47.7 to 49.0%. By 2030, An additional 70 million women are anticipated to be added by 2030 [1]. In 2019, among 1.9 billion women in the world who are in reproductive age (15–49 years), 842 million use contraceptives and 270 million still need contraceptives [2, 3]. From 2015 to 2020, the global proportion of family planning needs addressed by modern technologies remained stable at roughly 77%, but in the Africa region, it increased from 55 to 58% [4]. Contraception reduces the health risks associated with pregnancy for women, especially for adolescent girls, and when births are separated by less than two years, the infant mortality rate is 45% higher than it is when births are 2–3 years apart and 60% higher when births are four or more years apart [4].

The percentage of women of reproductive age who use modern methods of contraception to fulfill their need for family planning (SDG indicator 3.7.1) has gradually increased in recent decades, rising from 73.6 per cent in 2000 to 76.8% in 2020 [1]. There are several factors contributing to this gradual rise, including: a limited selection of procedures; limited access to services, especially for young, less fortunate, and single persons; worry or experience with adverse effects; and cultural or religious hostility; poor quality of available services; users’ and providers’ bias against some methods; and gender-based barriers to accessing services. As these barriers are addressed in some regions there have been increases in demand satisfied with modern methods of contraception.

The oral EC (emergency contraceptive) have various degrees of protection against pregnancy according to the time that they were taken. If it is taken within 72 h of unprotected sexual intercourse, they reduce the risks of pregnancy by 95%. Oral pills and IUCDs are mainly used as EC 72 h after sexual contact. After unprotected sexual activity, after sexual abuse, when regular contraception has been missed, or when contraception has not been used, EC is recommended [5].

Every year on average about 210 million throughout the world became pregnant. Of the 40–50 million women have abortion, 30 million occurred in developing nations. 20 million of the 40–50 million abortions carried out each year throughout the world are believed to be unsafe [6].

Unwanted pregnancy leading to unsafe abortion is most important causes of maternal morbidity and mortality. Globally, about 40% of the pregnancies (85 million) were unintended [7]. Women with unintended pregnancy may face the dilemma between terminating the pregnancy or allowing unwanted birth [8]. Every year, in developing countries, at least 22,000 women die from abortion-related complications [9]. In 2014, in Ethiopia 620,300 induced abortions were performed [10]. Abortion can hurt, even when done safely, and has psychological effects and physical stress for the women [11, 12].

In Ethiopia, unsafe abortion is a major public health problem. The country has a high incidence of unwanted pregnancies and incomplete and unsafe/ septic abortions, particularly among adolescents. Several studies showed that women who frequently have induced abortions in this country are less than 30 years old. Although the Ethiopian government works to prevent unintended pregnancies and abortion among young people under 24 years old, the number of young people seeking to end their pregnancies is rising each year [13]. One of the primary factors contributing to maternal morbidity and mortality in Ethiopian women is unsafe abortion due to unwanted pregnancy. Studies in the country showed that women who tend to undergo induced abortions are below the age of 30 and in higher educational level. Thus, they face a high risk of unwanted pregnancy [14]. Every year, in developing countries, at least 22,000 women dies from abortion-related complications [12]. An estimated 620,300 induced abortions were performed in Ethiopia in 2014 [10]. Even if performed safely, abortion is painful and might have psychological and physical stress [11, 12].

Various studies were conducted on knowledge and associated factors on EC utilization; however, little is known about the knowledge and extent of factors associated with emergency contraceptives utilization among female students in Ethiopia who are in reproductive age in College of Education. Therefore, the aim of this study is to assess knowledge about EC and factors associated with utilization of emergency contraceptive among female students in Bonga College of education.

## Methods

### Study area

This study was conducted in Bonga College of Education. Bonga town is located at 450 km from Addis Ababa. Bonga College of Education is one the oldest teacher education college in Ethiopia. Bonga college of education is located with geographical coordinates of 7°16’N and 36° 35’ E and an altitude of 1620 m above sea level (masl). In Bonga town EC services are available in Government health organization as well as in private health organizations.

### Study design

Cross-sectional study was conducted by recruiting female students in Bonga College of Education

### Study period

The study was conducted from November 10, 2022 to May 30, 2023.

### Study population

All female students recruited in this academic year were included in this study.

### Sample size and sampling procedure

All female students of Bonga Education College in all departments were included in this study purposively. A total of 554 students in both diploma and degree program enrolled in 2022/23 academic year. Among this 126 are females and 428 males in both programs. There are three active departments in diploma program and 12 in degree program. Since female students are few compared to male, all registered female students in both programs are included in this study.

### Data collection procedures

Data were collected using a self-administered Amharic version and a pretested questionnaire. It includes variables like socio-demographic characteristics, sexual history, knowledge about emergency contraception, risk factors, and practice on emergency contraceptive methods.

### Data analysis

Data obtained from the survey was entered into Microsoft Excel 2010 and analyzed with SPSS version 20.0. Data summary was done with descriptive statistics. Logistic regression test was used to measure associations between variables. Odds ratio was used to measure strengths of association between variables. Statistical significance was considered at 95% confidence level (CL). *P*-value less than 0.05 was considered significant during the analysis.

### Ethical consideration

To conduct this research, permission was obtained from Bonga College of Education (BCE) research and community service office. Informed written/verbal consent was obtained from female students. Orientation was given by the principal investigator using Amharic language and they were informed that their participation is on voluntary bases and that they can withdraw their consent at any time and then asked to put their signature on a consent form. Students were participated in the study were on voluntary basis after being informed about the purposes of the study and giving their consent. All the information obtained from each study participant was kept confidential.

### Operational definitions of terms

#### Attitude

People feelings towards emergency contraceptive.

#### Emergency contraceptive

A drug or device used after having unprotected sexual intercourse to avoid pregnancy.

#### Knowledge

Information that females have related to emergency contraceptives.

#### Poor knowledge

Respondents who scored above the mean.

#### Good knowledge

Respondents who scored below the average.

## Results

### Socio-demographic characteristics of respondents

In this study, a total of 103 female students participated. The mean age of the respondents was 20.6 (SD ± 2.06) years. The youngest being 18 and the oldest 30 years old. Among the total participants, 39 (37.9%) came from urban areas and 64 (62.1%) in rural areas. The majority of respondents are unmarried (83.5%) and others are married (14.6%) and some are divorced (1.9%). Majority of the participants are protestant faith follower (51.5%) followed by Orthodox Christian follower (42.7%. Muslim (1%) and Catholic (1%) faith follower are in small proportion. The remaining 3.9% are other faith follower.

### Respondent’s sexual history

Among the 103 female students, 31 (31.1%) had started sexual intercourse. The majority (58.1%) stared sex at the age of 13–19 years and others (41.9%) at the age of 20–25 years. About 58.1% respondents faced pregnancy. Majority of them (55.6%) experienced pregnancy one tomes, 27.8% two times and 16.7% three and above. The Majority of pregnancy (83.3%) was intended type whereas 16.7% was unwanted pregnancy. Among the total sexually experienced respondents, 93.5% use contraceptive methods while others 6.5% do not use.

### Knowledge about contraceptive methods

Among the total 103 female college students, 66(64.1%) heard about emergency contraceptives. Regarding the general knowledge about contraceptive methods, 19(18.4%) had poor knowledge of contraceptive methods. Mostly female students get information from school club, friends, school teachers, hospitals and family (Table 1). Only 35.9% and 7.8% respondents correctly identified the recommended time limit (that means within 72 h. for pills and within 120 h for IUCD) respectively. Female students get EC from pharmacy (12.6%), government health institutes (16.5%) and private clinics (3.9%).

**Table 1:** Respondents’ knowledge about contraception in Bonga College of education, Ethiopia, 2023

Variables	Frequency	Percent
General knowledge about contraceptive methods		
Poor	19	18.4
Good	17	16.5
Very good	15	14.6
Excellent	52	50.5
Source of information about contraceptive methods		
Family	16	15.5
Teachers	16	15.5
School clubs	19	18.4
Pharmacy	5	4.9
Clinic	6	5.8
Hospital	17	16.5
Friends	17	16.5
Others	7	6.8
Currently used EC		
Post pills	19	18.4
IUD	2	1.9
Nothing	4	3.9
Heard about EC		
Yes	66	64.1
No	31	30.1
Use EC		
Yes	27	87.1
No	4	12.9
Factors to use EC		
Forced sex	30	29.1
Condom failure	1	1.0
Missing regular pills	7	6.8
Free sex	15	14.6
I don’t know	40	38.8
Currently used birth control method		
Post pill	7	6.8
Regular pills	5	4.9
Injection	19	18.4
Condom	1	1.0
Norplant	3	2.9
Calendar method	5	4.9
None	56	54.4
Place to get EC		
Pharmacy	13	12.6
Gov health institute	17	16.5
Private clinic	4	3.9
All	32	31.1
I don`t know	32	31.1
Recommended time to take EC pills		
Any time	7	6.8
Within 72 h	37	35.9
Within 5days	1	1.0
I don`t know	50	48.5
Recommended time to take IUCD as EC		
Within 24 h	9	8.7
Within 48 h	2	1.9
Within 120 h	8	7.8
I don`t know	76	73.8

### Risk of unplanned pregnancy and factors associated to use emergency contraceptives

As presented in Table 2, abortion was a challenge for female students. A total of 4 (12.9%) female students had practiced abortion. Abortion takes place mainly in private clinics compared to government health institutes and others. Fear of discontinuing school was the main inducing factor to commit abortion. Unprotected free sex was the dominant cause to have unintended pregnancy. In fear of unwanted pregnancy female students use post pills as emergency pills rather than other contraceptive methods. Students use contraceptive pills and hormonal injections as general birth control methods. Forced sex (29.1%) and unprotected free sex (14.6%) are factors that urge female students to use emergency contraceptives.

**Table 2:** Unplanned pregnancy in female students and associated risks in Bonga College of education, Ethiopia, 2023

Variables	Frequency	Percent
Abortion experience		
Yes	4	12.9
No	27	87.1
Place where abortion takes place		
Private clinic	4	3.9
Government health institute	-	-
Self	-	-
Village traditional abortion	-	-
Reasons for induced abortion		
Fear of discontinuing school	4	3.9
Fear of parents	-	-
Economic problem	-	-
Since unintended	-	-
Fear of stigma	-	-
Action taken when unplanned pregnancy occurred		
Abort	4	12.9
Give birth	18	58.1
Difficult to decide	9	29
Reasons for unintended pregnancy		
Unprotected sex	9	8.7
Forced sex	1	1.0
In availability of birth control methods	1	1.0
Interest	1	1.0

### Determinants of emergency contraceptive utilization

The logistic regression analysis showed college female students whose age category above 25 years were more likely to use emergency contraceptives. Students who came from urban area are more likely to use EC than rural areas. Married female students (AOR = 2.5, 95% CI: 0.76, 8.7) were two times likely to use EC as contraceptive methods options. There was no statistically significant difference between different knowledge level about contraceptive methods and use of EC. Female students who had free sex used EC more likely (Table 3).

**Table 3:** Logistic regression analysis on emergency contraceptive utilization among female college students in Bonga College of education, Southwest Ethiopia, 2023

Variables	EC used		Crude OR (95% CI)	Adjusted OR (95% CI)	*P*-value (AOR)
Age group (in years)	Yes n (%)	No n (%)			
13–19	2 (7.4)	24 (31.6)	1.00	1.00	
20–25	24 (88.9)	50 (65.8)	0.04(0.002,3.7)	0.03(0.001,3.6)	*0.15*
Above 25	1 (3.7)	2 (2.6)	0.02(0.003,0.75)	0.01(0.001,0.6)	0.02*
Residence					
Urban	13(48.1)	26 (34.2)	1.00	1.00	
Rural	14(51.9)	50(65.8)	0.46(0.23,1.2)	0.36(0.13,1.0)	0.04*
Marital status					
Unmarried	18 (66.7)	68 (89.5)	1.00	1.00	
Married	8 (29.6)	7 (9.2)	2.6(0.82,9.1)	2.5(0.76,8.7)	*0.12*
Divorced	1 (3.7)	1 (1.3)	1.2(0.04,20.8)	1.0(0.02,20.5)	*0.6*
Unintended pregnancy					
Yes	10 (83.3)	5 (83.3)	3.8(0.5,45.3)	3.7(0.3,44.6)	*0.3*
No	2(16.7)	1(16.7)	1.00	1.00	
Knowledge of CM					
Poor	4(14.8)	15(19.7)	1.00	1.00	
Good	4(14.8)	13(17.1)	0.98(0.25,4.0)	0.92(0.22,3.69)	*0.9*
Very good	4(14.8)	11(14.5)	1.7(0.47,5.6)	1.4(0.37,5.3)	*0.6*
Excellent	15(55.6)	37(48.7)	1.0(0.32,3.7)	0.93(0.23,3.6)	*0.9*
Factors to use EC					
Forced sex	8(30.8)	22(32.4)	0.45(0.08,1.3)	0.35(0.09,1.27)	*0.11*
Condom failure	0(0.0)	1(1.5)			*1.00*
Missing regular pills	4(15.4)	3(4.4)	0.19(0.04,1.3)	0.18(0.02,1.2)	*0.08*
Free sex	9(34.6)	6(8.8)		0.09(0.02,0.4)	0.001*
I don`t know	5(19.2)	36(52.9)	1.00	1.00	

## Discussion

This cross-sectional study was conducted to determine the female students’ knowledge about emergency contraceptives and associated factors in Bonga College of Education. Among female students who had started sexual intercourse, 58.1% respondents faced pregnancy and the majority of pregnancy was intended type whereas 16.7% was unwanted pregnancy. Among the total sexually experienced respondents, 93.5% use different contraceptive methods while others 6.5% do not use. Similar studies showed higher tendency of using contraceptive method in this sexually active age [15–18].

This study showed that 26.2% female students used EC. Several studies on EC utilization in Ethiopia showed that female students used EC [15–17, 19–22]. The age group 20 to 25 year age use EC more likely [15, 19, 23].

Residence was an important determining factor for EC use. In this study college female students who came from rural areas were not using more likely emergency contraceptives as compared with those in the urban areas. This is consistent with the study done in Dessie Town showing that urban dweller uses EC more likely than rural [19]. This might be due to better expose to media which create awareness about birth control methods in urban areas compared to rural areas.

In this study 64.1% female students heard about EC and 18.4% had poor knowledge of contraceptive methods. There was significant association between respondents having good knowledge of contraceptive methods and EC utilization. Female students who had good and excellent knowledge on contraceptive methods were more likely to use emergency contraceptives as compared to those having poor knowledge. This finding was similar with studies conducted in several in Ethiopia [15–17, 19].

This study showed that forced sex (29.1%) and free unprotected voluntary based sex (14.6%) urge College female students to use emergency contraceptives. Similar studies in Ethiopia and Ghana reported unprotected sex as a major determinant factor [15, 20, 24].

This study presented the association between abortion inducing factor and places to be done. College female students did abortion in private clinic dominantly compared to other health institutions. Fear of discontinuing school was the main determinant factor to commit abortion. Similar study also reported in Debre Tabor town [15].

### Conclusion and recommendation

Higher proportion of students at age group of 20-to-25-year use EC more than other age groups.

Female students who came from urban area use EC than who came from rural areas. Majority of sexually active students had good practice and knowledge of using birth control method but some had poor knowledge of Contraceptive methods. Forced sex and free sexual practice are key determinant factors that urge to use EC. Abortion is a major health challenge reported which is done in private clinic mostly. Fear of discontinuing school was determinant factors identified to commit abortion.

Therefore, to improve female students’ reproductive health information related to emergency contraceptive and other general birth control methods, basic information and trainings on RH should be given to female students in the college.

## Data Availability

The datasets used and analyzed during the study are available from the corresponding Author on reasonable request.
